# Screening of a genome‐reduced *Corynebacterium glutamicum* strain library for improved heterologous cutinase secretion

**DOI:** 10.1111/1751-7915.13660

**Published:** 2020-09-06

**Authors:** Johannes Hemmerich, Mohamed Labib, Carmen Steffens, Sebastian J. Reich, Marc Weiske, Meike Baumgart, Christian Rückert, Matthias Ruwe, Daniel Siebert, Volker F. Wendisch, Jörn Kalinowski, Wolfgang Wiechert, Marco Oldiges

**Affiliations:** ^1^ Institute of Bio‐ and Geosciences – Biotechnology (IBG‐1) Forschungszentrum Jülich, Institute of Bio‐ and Geosciences ‐ Biotechnology (IBG‐1) Jülich 52425 Germany; ^2^ Bioeconomy Science Center (BioSC) Forschungszentrum Jülich Jülich 52425 Germany; ^3^ Microbial Genomics and Biotechnology Center for Biotechnology Bielefeld University Bielefeld 33615 Germany; ^4^ Faculty of Biology, Chair of Genetics of Prokaryotes Bielefeld University Bielefeld 33615 Germany; ^5^ Computational Systems Biotechnology (AVT.CSB) RWTH Aachen University Aachen 52074 Germany; ^6^ Institute of Biotechnology RWTH Aachen University Aachen 52074 Germany; ^7^ Present address: Institute of Microbiology and Biotechnology Ulm University Ulm 89081 Germany; ^8^ Present address: Microbial Biotechnology Campus Straubing for Biotechnology and Sustainability Technical University of Munich Straubing 94315 Germany

## Abstract

The construction of microbial platform organisms by means of genome reduction is an ongoing topic in biotechnology. In this study, we investigated whether the deletion of single or multiple gene clusters has a positive effect on the secretion of cutinase from *Fusarium solani pisi* in the industrial workhorse *Corynebacterium glutamicum*. A total of 22 genome‐reduced strain variants were compared applying two Sec signal peptides from *Bacillus subtilis*. High‐throughput phenotyping using robotics‐integrated microbioreactor technology with automated harvesting revealed distinct cutinase secretion performance for a specific combination of signal peptide and genomic deletions. The biomass‐specific cutinase yield for strain GRS41_51_NprE was increased by ~ 200%, although the growth rate was reduced by ~ 60%. Importantly, the causative deletions of genomic clusters cg2801‐cg2828 and *rrnC*‐cg3298 could not have been inferred *a priori*. Strikingly, bioreactor fed‐batch cultivations at controlled growth rates resulted in a complete reversal of the screening results, with the cutinase yield for strain GRS41_51_NprE dropping by ~ 25% compared to the reference strain. Thus, the choice of bioprocess conditions may turn a ‘high‐performance’ strain from batch screening into a ‘low‐performance’ strain in fed‐batch cultivation. In conclusion, future studies are needed in order to understand metabolic adaptations of *C. glutamicum* to both genomic deletions and different bioprocess conditions.

## Graphical abstract

The construction of microbial platform organisms by means of genome reduction is an ongoing topic in biotechnology. In this study, we investigated whether the deletion of single or multiple gene clusters has a positive effect on the secretion of cutinase from *Fusarium solani* pisi in the industrial workhorse *Corynebacterium glutamicum*. A total of 22 genome‐reduced strain variants were compared applying two Sec signal peptides from *Bacillus subtilis*. High‐throughput phenotyping using robotics‐integrated microbioreactor technology with automated harvesting revealed distinct cutinase secretion performance for a specific combination of signal peptide and genomic deletions. Strikingly, bioreactor fed‐batch cultivations at controlled growth rates resulted in a complete reversal of the batch screening results, with the cutinase yield for strain GRS41_51_NprE dropping by ~ 25% compared to the reference strain. Thus, the choice of bioprocess conditions may turn a “high‐performance” strain from batch screening into a “low‐performance” strain in fed‐batch cultivation.

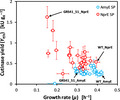

## Introduction

Investigating genome‐reduced strain (GRS) variants of important microbial production hosts for improved production performance is a current topic in biotechnology. In applied research, GRSs are often constructed in a top‐down approach by reducing the genome of a given organism to the necessary functionality with respect to given environmental constraints (Noack and Baumgart, 2019). This approach is considered useful for the construction of platform strains, which are well characterized and behave more predictably due to a less complex genome. Moreover, removing non‐essential genes dispenses with unwanted consumption of energy and metabolic building blocks, so that these resources are preserved for improved product yield (Hohmann *et al*., 2017).

The construction of streamlined chassis strains as configurable modules for industrial biotechnology development projects can be regarded as a technology driver for applied research (Juhas *et al*., 2014; Choe *et al*., 2016). Finally, it was postulated that the generated knowledge will facilitate the construction of functional cells from scratch for different purposes in a bottom‐up approach (Forster and Church, 2006). Genome reduction projects concerning industrially important microorganisms have been reported for *Bacillus subtilis* (Tanaka *et al*., 2013; Reuß *et al*., 2017), *Escherichia coli* (Pósfai *et al*., 2006), *Pseudomonas putida* (Lieder *et al*., 2015; Martínez‐García and Lorenzo, 2016) and *Corynebacterium glutamicum* (Baumgart *et al*., 2018). The latter project resulted in the chassis strain C1* with a genome reduction of 13.4%, while displaying wild‐type‐like growth behaviour and robustness with respect to different environmental conditions. In addition, a strain library with defined genomic deletions, resulting in various growth phenotypes, was made available for further systematic studies on *C. glutamicum*.

The use of *C*. *glutamicum* for heterologous protein secretion is attracting increasing interest (Freudl, 2017). This microbial cell factory exhibits favourable features with respect to secretory enzyme production, namely low nutritional demand, low amount of endogenously secreted proteins and proteolytic activity, as well as the ability to secrete heterologous proteins into the extracellular medium in the g l^‐1^ range (Watanabe *et al*., 2013; Liu *et al*., 2013a,b; Ravasi *et al*., 2015; Yim *et al*., 2016) via the Sec or Tat pathway (Freudl, 2017). Furthermore, since *C. glutamicum* has been a major producer for food and feed amino acids on an industrial scale for decades (Wendisch, 2014; Wendisch *et al*., 2016; Freudl, 2017), extensive bioprocess knowledge of this organism and methods for its genetic manipulation are already available. In addition, it can be cultivated to high cell densities (Knoll *et al*., 2007; Yim *et al*., 2013; Yim *et al*., 2016) and has been shown to be very robust with respect to process inhomogeneities encountered in large‐scale cultivations (Käß *et al*., 2014; Limberg *et al*., 2016; Limberg *et al*., 2017).

A frequent task in production strain engineering is the assignment of performance indicators, such as titre, yield and productivity, to newly constructed strain variants. This is often referred to as quantitative phenotyping (Hemmerich *et al*., 2019b). For such screenings of mutant strain libraries, microbioreactor (MBR) systems are ideally suited, as they provide elevated cultivation throughput with the option of tight environmental control of cultivation parameters (Lattermann and Büchs, 2015; Hemmerich *et al*., 2018). Typically, individual cultivations in MBR systems can be monitored closely with several online and at‐line analytics (Unthan *et al*., 2015b; Flitsch *et al*., 2016; Ladner *et al*., 2016; Cruz Bournazou *et al*., 2017). Defined and controllable cultivation conditions in MBRs allow the quantitative phenotyping of strain libraries. These aspects mean that MBRs are superior to simple microplate screenings. The ability to integrate MBR systems with an automated liquid handling system (LHS) greatly expands the strain library phenotyping capacities of MBR systems (Huber *et al*., 2009). In particular, automated sampling and harvesting procedures enable the acquisition of data allowing mutant strains that grow differently to be compared (Rohe *et al*., 2012). Importantly, (semi‐)automated data processing is needed to take full advantage of automated and integrated MBR systems (Neubauer *et al*., 2013; Hemmerich *et al*., 2018).

As yet there has been no systematic investigation of the interrelation of genome reduction in *C. glutamicum* and recombinant protein secretion imposing a considerable metabolic burden, the aim of this study was to screen for gene cluster deletions (and combinations thereof) that positively affect heterologous protein secretion. As maximizing productivity is interesting from an industrial perspective, a GRS showing improved protein secretion performance in batch screening mode should be additionally tested in stirred tank reactor (STR) fed‐batch cultivations, which is the typical mode of operation for industrial application (Riesenberg and Guthke, 1999).

## Results and discussion

In this study, 22 *C. glutamicum* GRSs (Unthan *et al*., 2015a; Baumgart *et al*., 2018) were screened for the secretion of heterologous cutinase of *Fusarium solani pisi* as a model hydrolase of eukaryotic origin. To account for possible interrelations between growth and protein secretion, the selection explicitly included strains with growth defects compared to the wild‐type strain. Cutinase is applied in, for example, detergents, and food and textile processing (Chen *et al*., 2013). To enable cutinase secretion via the Sec pathway, the plasmid‐encoded cutinase gene was fused to a Sec signal peptide (SP) sequence. Since the optimal SP for a certain target protein cannot be predicted *in silico* (Brockmeier *et al*., 2006; Mathiesen *et al*., 2009; Watanabe *et al*., 2009; Zhang *et al*., 2015), cutinase secretion with the *C. glutamicum* GRS library was tested using two Sec SPs (AmyE SP and NprE SP) of *B. subtilis* (Brockmeier *et al*., 2006), which show divergent cutinase secretion performance in *C. glutamicum* (Hemmerich *et al*., 2019a).

### Rapid quantitative phenotyping of a *C. glutamicum* GRS library with respect to growth and heterologous cutinase secretion

The resulting 44 cutinase secreting GRSs (22 strains, each with either AmyE SP or NprE SP) were assessed for growth rate (*µ*) and biomass‐specific cutinase yield (*Y*_P/X_) using an MBR system integrated with an LHS (Rohe *et al*., 2012). Here, cutinase activity in supernatants was measured as a proxy for the amount of functional cutinase secreted. Thus, biomass‐specific cutinase yield is expressed as secreted cutinase activity per biomass. Using the automated MBR approach, cultures showing strongly different growth behaviour can be harvested automatically under the same physiological conditions, in this case shortly after the transition from exponential to stationary growth phase. This automation approach for quantitative strain phenotyping allows a standardized comparison of cutinase secretion performance.

**Fig. 1:**
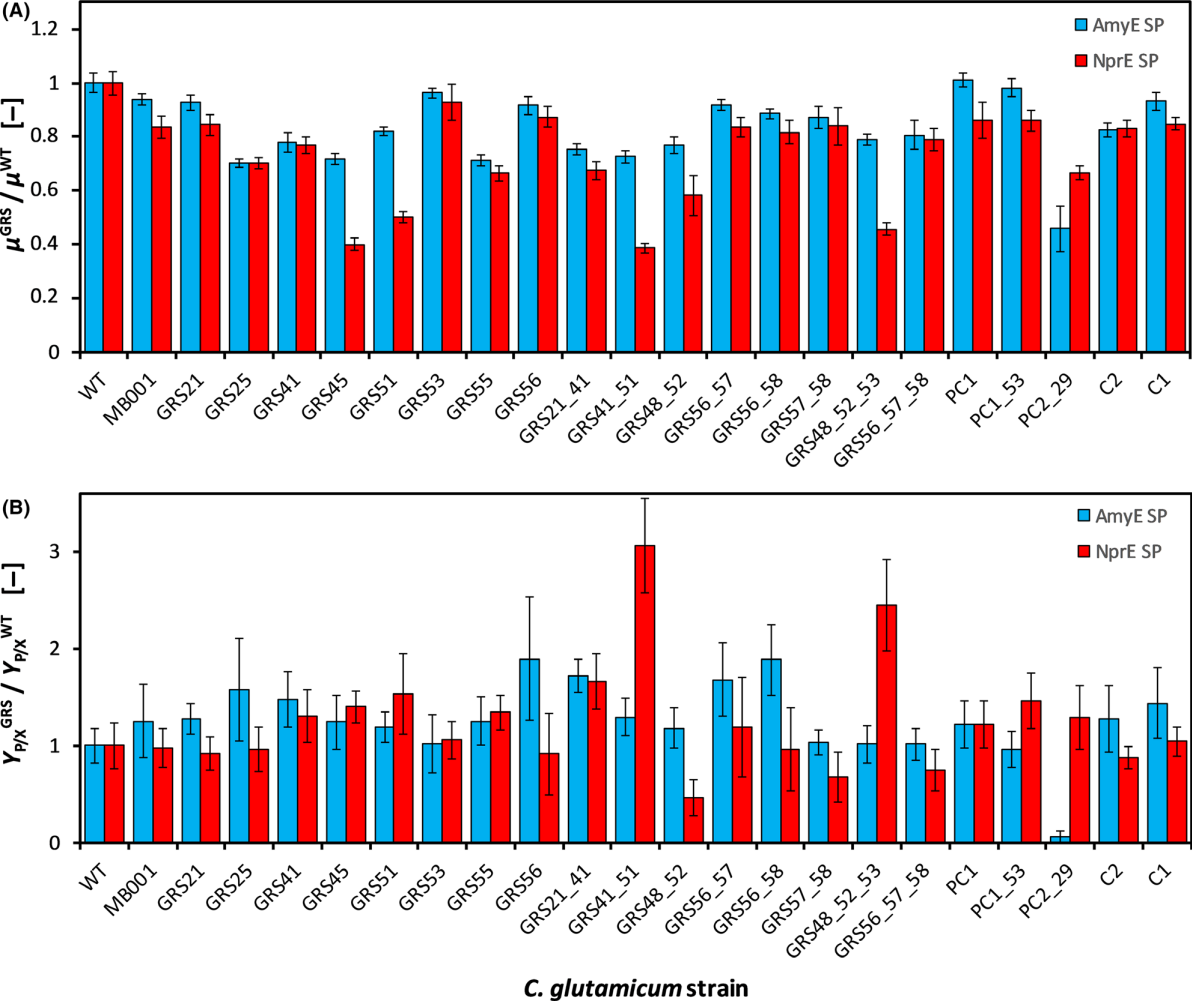
Observed growth rates *µ* and biomass‐specific cutinase yields *Y*_P/X_ from *C. glutamicum* GRS library screening, normalized with respect to phenotype of the respective reference strain. (A) Relative GRS‐specific growth rates *µ* ^GRS^/*µ* ^WT^. (B) Relative GRS‐specific cutinase yields *Y*_P/X_^GRS^/*Y*_P/X_^WT^. Each bar represents one GRS, and the Sec SP used is indicated by different colours. Error bars indicate standard deviation from growth experiments conducted in eleven to 46 replicates, as indicated in Table S1. GRSs were cultivated aerobically with 20 g l^−1^ d‐glucose in CGXII‐defined mineral medium, using an LHS with integrated MBR system. Cultivations took place in 48‐well FlowerPlates (800 µl culture volume, 30 °C, 1300 rpm). Cultivations were automatically harvested shortly after transition from exponential to stationary phase. *GRS*, genome‐reduced strain. *SP*, signal peptide. *LHS*, liquid handling system. *MBR*, microbioreactor.

Results from quantitative phenotyping of the GRS library for both AmyE SP‐ and NprE SP‐mediated cutinase secretion are found as absolute values in Table S1 and are shown as relative values with respect to the corresponding wild‐type strains in Fig. 1. The range of biomass‐specific cutinase yields obtained in batch cultivation for the AmyE and NprE SP was 0.01–0.45 and 0.37–1.62 kU g_X_^−1^ respectively. The range of observed growth rates was approximately 0.15–0.37 and 0.13–0.42 h^−1^ for the NprE SP and AmyE SP respectively. The slowest growing strain with NprE SP (GRS41_51_NprE) was identified as the top performer in terms of cutinase yield *Y*_P/X_. Strikingly, this was not the case for the slowest growing strain with AmyE SP (PC2_29_AmyE), for which hardly any secreted cutinase activity was detectable.

When comparing the observed growth phenotypes, it is seen that for many GRSs, the growth rate was reduced by not more than about 20% for both Sec SPs tested, i.e. irrespective of the Sec SP used (Fig. 1A). In most cases, the reduction in growth rate from WT_AmyE to GRS_AmyE was comparable to the reduction in growth rate from WT_NprE to GRS_NprE. For example, strain GRS41 showed the same growth rate reduction when using the AmyE SP or NprE SP, which was about 20 % less than the growth rate obtained with the corresponding reference strains (WT_AmyE or WT_NprE). The same observation was also made for, e.g., strain C2 as well as the *rrn* deletion strains GRS56, GRS56_57, GRS56_58, GRS57_58 and GRS56_57_58, whose growth rates were reduced by 10–30%, irrespective of the applied Sec SP.

Many of the strains displayed a cutinase yield that was comparable or only slightly different to the level of the reference strains WT_AmyE and WT_NprE. Interestingly, there were a few strains with a distinct growth phenotype when the two SPs were compared, as well as in comparison with the use of the respective SPs in the WT analogue (see Fig. 1A). For example, strain GRS45_AmyE showed a growth rate reduction of approximately 30%, while the same strain containing the NprE SP (i.e. strain GRS45_NprE) showed a growth rate reduction of approximately 60%. Similar patterns were also observed for strains GRS51_NprE, GRS41_51_NprE and GRS48_52_53_NprE (see also Table S1).

When comparing the GRS‐specific data shown in Fig. 1A and B, a relation between growth phenotype and cutinase secretion phenotype can be deduced for each GRS. In general, if an SP‐specific growth rate reduction was observed for a GRS, then the presence of the NprE SP induced a stronger growth burden compared to the AmyE SP, the only exception being for strain PC2_29. For this strain, the AmyE SP imposed a higher growth burden than the NprE SP (*μ* ^AmyE^ = 0.19 ± 0.03 h^−1^ vs. *μ* ^NprE^ = 0.26 ± 0.01 h^−1^). Notably, this growth defect was accompanied by an absence of extracellular cutinase activity (*Y*_P/X_^AmyE^ = 0.01 ± 0.01 kU g_X_^−1^ vs. *Y*_P/X_^NprE^ = 0.69 ± 0.17 kU g_X_^−1^). Two strains showed an exceptionally high cutinase yield: GRS48_52_53_NprE and GRS41_51_NprE (~ 2.5‐fold and ~ 3.1‐fold compared to WT_NprE, respectively, see Fig. 1). Interestingly, the two strains did not share any genomic deletions except ΔCGP123 and ΔIS*Cg*12, which all other GRSs shared as well. Apparently, different genomic deletions were responsible for increased cutinase yield when targeting the secretory pathway via the NprE Sec SP.

For GRS48_52_53_NprE, it can be concluded that its observed phenotype of slow growth and high cutinase yield was caused by the combinatorial loss of at least two genes located within the clusters cg3263‐cg3301 and cg3324‐cg3345. This result was obtained from the differential phenotyping of closely related GRSs (Hemmerich *et al*., 2019b). Moreover, the absolute values of growth rate and cutinase yield determined in this study for GRS48_52_53_NprE agree very well with previous results (Hemmerich *et al*., 2019b).

However, the highest cutinase yield with respect to both absolute and relative values was achieved with strain GRS41_51_NprE. These findings mean that this strain is the well‐reasoned choice out from GRS library for further investigation.

### Deletion of both cg2801‐cg2828 and *rrnC*‐cg3298 dramatically improved cutinase yield in combination with NprE SP

**Fig. 2:**
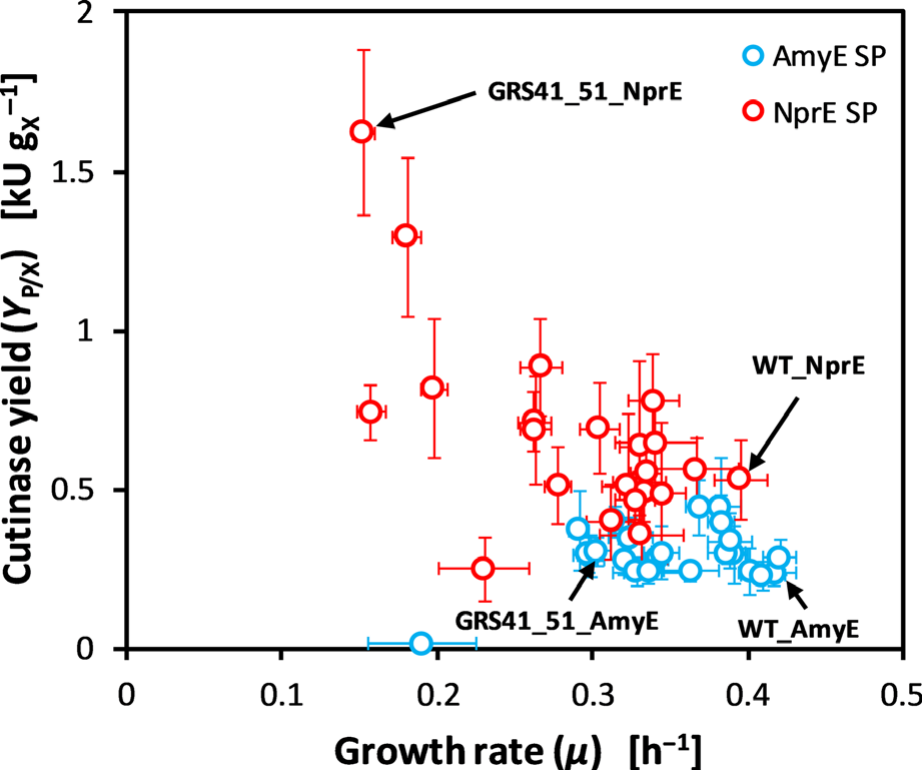
Correlation of biomass‐specific cutinase yields *Y*_P/X_ with growth rates *µ* for the cutinase secretion GRS library. The overall best performing strain GRS41_51_NprE is indicated, as well as its counterpart using the AmyE SP for cutinase secretion. For comparison, the corresponding reference strains WT_NprE and WT_AmyE are also indicated. Each data point represents one GRS, and the Sec SP used is indicated by different colours. Error bars indicate standard deviation from growth experiments conducted in eleven to 46 cultivation replicates, as indicated in Table S1. *GRS*, genome‐reduced strain. *SP*, signal peptide.

The negative correlation between cutinase yield *Y*_P/X_ and growth rate *µ* for strains GRS41_51_NprE and GRS41_51_AmyE in direct comparison to the other strains is seen in Fig. 2. The corresponding reference strains WT_NprE and WT_AmyE are also indicated.

Interestingly, the strongly affected phenotype of strain GRS41_51_NprE in comparison with WT_NprE with respect to growth rate as well as to cutinase yield was only seen for the very specific combination of Δcg2801‐cg2828, Δ*rrnC*‐3298 and NprE SP, and comparably pronounced phenotypes were not observed for strain GRS41_51_AmyE. Since almost all other GRSs showed higher cutinase yield with the NprE SP than the AmyE SP, and in accordance with previous studies (Hemmerich *et al*., 2019a,b), the strong positive effect of the NprE SP was easily anticipated. Based on the observation that deleting gene clusters cg2801‐cg2828 and *rrnC*‐cg3298 caused a strong growth defect in CGXII medium (Unthan *et al*., 2015a), this strain would not have been the first choice as a potential secretion host.

**Fig. 3:**
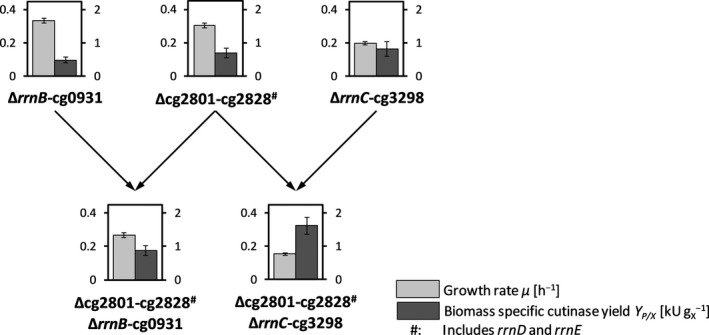
Impact of single gene cluster deletions (upper row) and combinations thereof (lower row) on growth and cutinase secretion phenotypes for resulting GRSs. Subscripts of bar plots indicate relevant single gene cluster deletion of strains GRS21_NprE, GRS41_NprE and GRS51_NprE (upper row, from left to right), as well as combinatorial deletion of gene clusters for strains GRS21_41_NprE and GRS41_51_NprE (lower row, left and right respectively). For strain GRS41_51_NprE, the specific combination of deleting clusters cg2801‐cg2828 and rrnC‐cg3298 led to the strongly increased biomass‐specific cutinase yield. This specific combination was not obvious from the other data obtained. Strains are based on CR099; for details, see Table 1. Bar plots show growth rate *µ* (light grey) and biomass‐specific cutinase yield *Y*_P/X_ (dark grey); for corresponding values, see Table S1. *GRS*, genome‐reduced strain.

In Fig. 3, the genetic relationships for GRS41_51_NprE and its predecessors are compared to the respective observed growth rates and biomass‐specific cutinase yields, as determined from MBR screenings. It was obvious that none of the other strains showed a prominent cutinase yield thus pointing to an interesting gene cluster deletion in this sense. Apparently, with respect to growth rates, the deletion of gene clusters including *rrn* operons caused growth defects, though to different extents (Δ*rrnB*‐cg0931: −16% for GRS21_NprE; Δcg2801‐cg2826 (includes *rrnD* and *rrnE*): −23% for GRS41_NprE; and Δ*rrnC*‐cg3298: −50% for GRS51_NprE).

Surprisingly, the combinatorial deletion of two out of the three clusters only greatly affected cutinase yield in a positive sense for strain GRS41_51_NprE. This indicates that the combined loss of at least one gene in cluster cg2801‐cg2828 and one gene in cluster *rrnC*‐cg3298 should be considered as the primary cause of the observed remarkable increase in cutinase yield. More specifically, the concerted loss of at least two genetic functions (found in different clusters) resulted in the observed drastic changes of phenotypes. At the moment, it cannot be concluded exactly which gene deletions were the root cause. An overview of deleted genes for GRS41_51_NprE with corresponding annotations is given in Table S2. As the deletion of two operons encoding of ribosomal RNA was reported not to affect growth (Unthan *et al*., 2015a), the additional deletion of a third *rrn* operon (*cgr10* – *cgr12*) apparently induced a reduced growth rate, probably due to a reduced ribosomal capacity in the cell. Contrary, it remains unclear how this would positively impact heterologous cutinase secretion. One may speculate that the loss of cg2804 encoding for a transposase may be involved, but for strain GRS51_NprE with two transposases and one putative transposase deleted (cg3266, cg3278, cg3296‐cg3298), no increased cutinase secretion performance was observed. Apparently, the concurrently observed strongly reduced growth rate seemed to be a necessary condition to achieve a high cutinase yield, as a comparable observation was made for strain GRS48_52_53_NprE (Hemmerich *et al*., 2019b).

For *P. putida*, an improved growth and biomass‐specific intracellular GFP yield were reported in bioreactor batch cultivations. Deleted genes encoded for energy‐costly functions or were known to enhance genetic stability (Lieder *et al*., 2015). Previously, it was shown that intracellular protein production in *C. glutamicum* can be increased by deleting genes encoding for prophages containing a restriction–modification system, resulting in a genome reduction of 6% (Baumgart *et al*., 2013). In contrast, the results from this study indicate that genome reduction as a tool for improved secretory production is more complex to adjust and less well understood in comparison with intracellular protein production.

### Cutinase secretion performance of GRS41_51_NprE in glucose‐limited, growth rate‐adjusted fed‐batch cultivations

**Fig. 4:**
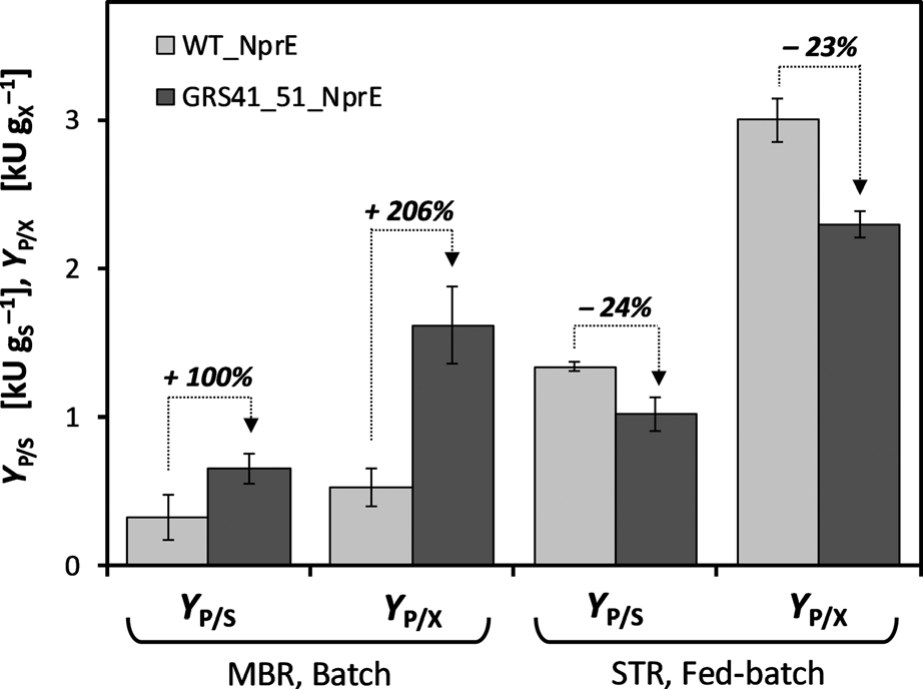
Effect of bioprocess conditions on substrate‐specific cutinase yield *Y*_P/S_ and biomass‐specific cutinase yield *Y*_P/X_ for strain GRS41_51_NprE and reference strain WT_NprE. Arrows indicate relative changes in parameter values from strain WT_NprE (light grey bars) to GRS41_51_NprE (dark grey bars). Data for fed‐batch cultivations with WT_NprE are taken from the literature (Hemmerich *et al*., 2019a). Bioreactor fed‐batch cultivations were conducted using a glucose feeding function calculated with feeding profile parameter *µ*_Set_ = 0.15 h^−1^. IPTG to induce recombinant gene expression was added to a final concentration of 50 µM at the beginning of the fed‐batch phase. *MBR*, microbioreactor. *STR*, stirred tank reactor.

In general, glucose‐limited fed‐batch cultivations are favourable in preference to maximum growth rate batch cultivations for heterologous protein production with various microorganisms (Yee and Blanch, 1992; Riesenberg and Guthke, 1999; Looser *et al*., 2015). In particular, this was recently confirmed for heterologous cutinase secretion with *C. glutamicum* (Hemmerich *et al*., 2019a). Therefore, strain GRS41_51_NprE was subjected to cutinase secretion performance evaluation in fed‐batch cultivations using an STR system on a laboratory scale. Exponential feeding rates in STR fed‐batch cultivations were adjusted to yield a growth rate of the culture (*µ*_Exp_ = 0.12 ± 0.01 h^−1^) slightly below the maximum growth rate of GRS41_51_NprE (*µ*_max_ = 0.15 ± 0.01 h^−1^), obtained from glucose‐unlimited MBR batch cultivations. This ensured continuously glucose‐limited, exponential growth. For comparison, data for strain WT_NprE obtained under the same bioprocess conditions are taken from the literature (Hemmerich *et al*., 2019a). The performance of strain GRS41_51_NprE in comparison with the reference strain WT_NprE is shown in Fig. 4, emphasizing the different bioprocess conditions that can be realized in the different cultivation systems (MBR and STR).

With respect to growth rate, GRS41_51_NprE showed no significant difference to WT_NprE, as expected, since the growth rate was determined by the exponential feed profile during the glucose‐limited fed‐batch phase (data not shown). Most strikingly, and in contrast to the results from MBR batch screenings, GRS41_51_NprE could not compete with WT_NprE with respect to cutinase yield *Y*_P/X_ in the fed‐batch process. More specifically, cutinase yield for GRS41_51_NprE was reduced by 23% compared to WT_NprE, while in MBR batch screenings this value was increased by 206%. A similar pattern was observed with respect to substrate‐specific product (cutinase) yield *Y*_P/S_, which indicates the efficiency of substrate conversion into the desired product. The data show that in MBR batch cultivations, strain GRS41_51_NprE converted glucose substrate more efficiently into cutinase product than the reference strain WT_NprE (*Y*_P/S_ was increased by 100%). Strikingly, in glucose‐limited fed‐batch cultivations, the opposite is seen. Strain GRS41_51_NprE showed a lower efficiency in terms of substrate‐to‐product conversion (i.e. *Y*_P/S_ was reduced by 24%). However, absolute values for *Y*_P/X_ and *Y*_P/S_ were higher in growth‐limited fed‐batch cultivations than in substrate non‐limited MBR batch screenings for both strains. The 14% genome‐reduced *E. coli* MDS40 was reported to show comparable behaviour in terms of growth and intracellular recombinant protein formation to its parental strain MG1655 in fed‐batch cultivations at controlled growth rates (Sharma *et al*., 2007). As is apparent from the results of this study, the desirable secretion of a heterologous target protein introduces another layer of complexity for designing bioprocesses with engineered production hosts.

Obviously, the availability of glucose, i.e. its growth‐limiting supply in fed‐batch versus non‐limiting excess conditions in batch mode, strongly determined cutinase secretion performance of *C. glutamicum* strains. In particular, the superior cutinase secretion performance of GRS41_51_NprE in MBR batch cultivations was characterized by a higher efficiency in both substrate‐to‐product conversion (i.e. increased *Y*_P/S_) and a higher product selectivity of the cell mass (i.e. increased *Y*_P/X_). When grown under continuous growth‐limiting glucose supply using a fed‐batch bioprocess, strain GRS41_51_NprE was less efficient in terms of cutinase secretion performance than the reference strain WT_NprE, as indicated by a reduction of 24% and 23% for *Y*_P/S_ and *Y*_P/X_ respectively (see Fig. 4). Apparently, *C. glutamicum* flexibly adapts to the bioprocess conditions it experiences, resulting in a sensitive response with respect to cutinase secretion performance.

The results suggest that the ranking obtained for a strain library is specific for the bioprocess conditions applied to evaluate the respective strain library. This implies such a ranking would be different for different bioprocess conditions. Similar to previous results, showing that the choice of bioprocess conditions can turn a ‘good’ Sec SP into a ‘bad’ one (Hemmerich *et al*., 2019a), it is seen in this study that the choice of bioprocess conditions can turn a ‘high‐performance’ strain from batch screening into a ‘low‐performance’ strain when applied in fed‐batch cultivation. In conclusion, the metabolism of *C. glutamicum* adapts to both the genomic deletions and different bioprocess conditions in an unpredictable manner for reasons to be identified in future studies.

## Conclusions

Previously, the deleted gene clusters in this study have been characterized with respect to growth in defined CGXII medium. Screening of a *C. glutamicum* GRS library with the additional objective of heterologous cutinase secretion revealed surprising phenotypes in terms of growth and cutinase secretion that could not have been inferred *a priori*. Strikingly, it makes a huge difference with respect to strain phenotypes whether growth is controlled by internal factors (such as genomic deletions) or external factors (such as fed‐batch profile). This suggests that there are unknown interactions derived from expression products of deleted genes rendering the experimental validation of GRS variants indispensable. The resulting rapidly increasing number of possible strain variants requires screening workflows with increased throughput under well‐defined conditions. In addition, this study clearly shows that screening results from high‐throughput quantitative phenotyping batch workflows need to be carefully revalidated with respect to fed‐batch bioprocess conditions.

This highlights the current dilemma in strain and bioprocess engineering, especially for the interrelation between these two typical tasks in biotechnological production process development. The vast number of strain variants which are easily obtainable by modern methods in genetic engineering cannot be characterized in fully controllable bench‐scale bioreactors for each type of bioprocess. If the final envisaged bioprocess operation mode is known, it is advisable to mimic it as much as possible in strain phenotyping workflows. If the bioprocess operation for production scale is not fixed in advance, it can be assumed that proper selection of the ‘best’ production strain is a crucial, non‐trivial task in the bioprocess development workflow.

## Experimental procedures

Unless otherwise stated, all chemicals were of analytical grade and purchased from Sigma, Merck or Roth. Data processing was conducted with Microsoft Excel (vers. 2010, 2016) and MATLAB with Statistics Toolbox (vers. 2013a to 2017b).

### Cultivation media, strain construction and strain maintenance

All strains and plasmids used in this study are listed in Table 1. Cultivations were conducted with BHI medium containing 37 g l^−1^ brain heart infusion broth, BHIS medium (BHI with additional 91 g l^−1^ sorbitol) and defined CGXII medium (Keilhauer *et al*., 1993) with 30 mg l^−1^ protocatechuic acid. The construction of deletion mutants was performed by double homologous recombination as described previously (Unthan *et al*., 2015a; Baumgart *et al*., 2018). All plasmids used for the construction of new mutants are listed in Table 1. Oligonucleotides used for the construction of new deletion plasmids are listed in Table S3. The deletion plasmids were constructed by overlap extension PCR or Gibson assembly as described previously (Unthan *et al*., 2015a; Baumgart *et al*., 2018). The deletion of *rrn* clusters was verified by shotgun sequencing using the Nextera DNA Sample Preparation Kit (Illumina) for library preparation and the Illumina MiSeq platform for sequencing of 2 × 300 bp (paired‐end reaction). Transformation was done by electroporation (van der Rest *et al*., 1999). To confirm cutinase secretion, transformed cell material was plated on indicator agar plates containing 100 µM IPTG to induce cutinase secretion and Tween‐20 (1% v v^−1^) which is hydrolysed by cutinase, visible by halo formation. From each transformation, a single colony was spread on a new agar plate to generate a sufficient amount of isogenic cell mass, which was resuspended into freezing solution (1 volume phosphate‐buffered saline (PBS; 8 g l^−1^ NaCl, 0.2 g l^−1^ KCl, 1.78 g l^−1^ NaH_2_PO_4_ ∙ 2 H_2_O, 0.27 g l^−1^ K_2_HPO_4_, pH 7.4 ± 0.1) and 1 volume of 500 g l^−1^ glycerol solution) and stored in 2 ml aliquots at −80 °C as the master cell bank (MCB). A few 100 µl from an MCB aliquot was used to inoculate an overnight shake flask culture with 50 ml CGXII medium containing 10% (v v^−1^) BHI medium and, if appropriate, 25 mg l^−1^ kanamycin. After growth to saturation, one volume of the cell suspension was combined with 1 volume of glycerol solution (500 g l^−1^) and aliquots were stored at −80 °C as the working cell bank (WCB).

**Table 1:** *Corynebacterium glutamicum* strains and plasmids used in this study.

Name	Relevant characteristics	Reference
*Strains*		
WT	ATCC 13032 wild‐type strain	Kinoshita *et al*. (1957)
MB001	WT ΔCGP123 (Δcg1507‐cg1524 Δcg1746‐cg1752 Δcg1890‐cg2071)	Baumgart *et al*. (2013)
CR099	MB001 ΔIS*Cg*12	Baumgart *et al*. (2013)
GRS21	CR099 Δ*rrnB*‐cg0931	Unthan *et al*. (2015b)
GRS25	CR099 Δcg1281‐cg1289	Unthan *et al*. (2015b)
GRS41	CR099 Δcg2801‐cg2828	Unthan *et al*. (2015b)
GRS45	CR099 Δcg2990‐cg3006	Unthan *et al*. (2015b)
GRS51	CR099 Δ*rrnC*‐cg3298	Unthan *et al*. (2015b)
GRS53	CR099 Δcg3324‐cg3345	Unthan *et al*. (2015b)
GRS55	CR099 Δcg3000‐cg3006	This study
GRS56	CR099 Δ*rrnB*	This study
GRS21_41	CR099 Δcg2801‐cg2828 Δ*rrnB*‐cg0931	Unthan *et al*. (2015b)
GRS41_51	CR099 Δcg2801‐cg2828 Δ*rrnC*‐cg3298	Unthan *et al*. (2015b)
GRS48_52	CR099 Δcg3102‐cg3111 Δcg3263‐cg3301	Baumgart *et al*. (2018)
GRS56_57	CR099 Δ*rrnB* Δ*rrnC*	This study
GRS56_58	CR099 Δ*rrnB* Δ*rrnF*	This study
GRS57_58	CR099 Δ*rrnC* Δ*rrnF*	This study
GRS48_52_53	CR099 Δcg3102‐cg3111 Δcg3263‐cg3301 Δcg3324‐cg3345	Baumgart *et al*. (2018)
GRS56_57_58	CR099 Δ*rrnB* Δ*rrnC* Δ*rrnF*	This study
PC1	CR099 Δcg2312‐cg2322 Δcg2621‐cg2643 Δcg2663‐cg2686 Δcg2755‐cg2760 Δcg3102‐cg3111	Baumgart *et al*. (2018)
PC1_53	PC1 Δcg3324‐cg3345	This study
PC2_29	CR099 Δcg0414‐cg0440 Δcg0635‐cg0646 Δcg0704‐cg0748 Δcg0822‐cg0845 Δcg1018‐cg1033 Δcg1172‐cg1213 Δcg1291‐cg1305 Δcg1340‐cg1353 Δcg1370‐cg1385	This study
C1	PC1 Δcg0635‐cg0646 Δcg0704‐cg0748 Δcg0822‐cg0845 Δcg1018‐cg1033 Δcg1172‐cg1213 Δcg1291‐cg1305	Baumgart *et al*. (2018)
C2	CR099 Δcg0414‐cg0440 Δcg0635‐cg0646 Δcg0704‐cg0748 Δcg0822‐cg0845 Δcg1018‐cg1033 Δcg1172‐cg1213 Δcg1291‐cg1305 Δcg1340‐cg1352 Δcg2312‐cg2322 Δcg2621‐cg2643 Δcg2663‐cg2686 Δcg2755‐cg2760 Δcg3072‐cg3091 Δcg3102‐cg3111	Baumgart *et al*. (2018)
*Plasmids*		
pEKEx2‐SP‐cutinase	Cutinase gene from *F. solani pisi* ligated to Sec SP sequence [*amyE*, *epr*, *nprE*, *ypjP* or *ywmC*] from *B. subtilis*, cloned into pEKEx2 plasmid under control of P_tac_, Kan^R^	Rohe *et al*. (2012)
pK18*mobsacB*	Kan^R^.; plasmid for allelic exchange in *C. glutamicum*; (pK18 *ori*V*_E.c_*_._, *sacB*, *lacZ*α)	Schäfer *et al*. (1994)
pK19*mobsacB*	Kan^R^.; plasmid for allelic exchange in *C. glutamicum*; (pK19 *ori*V*_E.c_*_._, *sacB*, *lacZ*α)	Schäfer *et al*. (1994)
pK19*mobsacB*Δcg3324‐cg3345	pK19*mobsacB* derivative for deletion of cg3324‐cg3345	Unthan *et al*. (2015b)
pK19*mobsacB*Δcg1370‐cg1385	pK19*mobsacB* derivative for deletion of cg1370‐cg1385	Unthan *et al*. (2015b)
pK19*mobsacB*Δcg3000‐cg3006	pK19*mobsacB* derivative for deletion of cg3000‐cg3006	This study
pK18*mobsacB*Δ*rrnB*	pK18*mobsacB* derivative for deletion of *rrnB*	This study
pK18*mobsacB*Δ*rrnC*	pK18*mobsacB* derivative for deletion of *rrnC*	This study
pK18*mobsacB*Δ*rrnF*	pK18*mobsacB* derivative for deletion of *rrnF*	This study

### Microbioreactor cultivations

For MBR screenings, each strain in this study was cultivated in three to sixteen parallel cultivations in a single MBR run, and the MBR runs were conducted in two to five replicates. The individual number of cultivations per strain is found in Table S1.

#### Pre‐culturing

For MBR cultivation experiments, two sequential pre‐cultures were conducted in shake flasks. The first pre‐culture was inoculated from a frozen WCB aliquot and cultivated in 15 ml BHI medium. After approx. 6 h, 300 µl from the first pre‐culture served as the inoculum for the second pre‐culture (50 ml CGXII medium, approx. 16 h incubation). Then, the second pre‐culture was centrifuged (4000 *g*, 5 min, 4 °C), resuspended in sterile PBS and used to inoculate the MBR culture to an initial OD_600_ of approximately 0.2.

#### Main cultivations

Main cultivations were carried out in an MBR device with 48 flower‐shaped well microplates (BioLector and FlowerPlate, m2p‐labs, Baesweiler, Germany), integrated in an LHS (Rohe *et al*., 2012). Cultivation conditions were as follows: CGXII medium with 20 g l^−1^ d‐glucose, 800 µl per well, 1300 rpm at a shaking diameter of 3 mm, 30 °C. To induce cutinase production, 100 µM IPTG was added. The integrated BioLector devices are capable of quasi‐continuous monitoring of biomass concentration via backscatter measurements (Kensy *et al*., 2009), as well as pH and dissolved oxygen (DO) via fluorescence sensor spots (optodes) integrated at the bottom of each cultivation well. FlowerPlates were covered with gas‐permeable sterile sealing foils suitable for robotic access to the culture (F‐GP‐10 and F‐GPRS48‐10, m2p‐labs, Baesweiler, Germany).

#### Automated harvesting procedure

Automated harvesting and sampling procedures were based on pre‐defined triggers, which utilize the MBR process data monitored online. Triggers are defined using a supervising software (RoboLector Agent, m2p‐labs, Baesweiler, Germany), which activates the integrated LHS to act on the individual MBR cultures by writing pipetting lists (handshake files). The LHS device runs a program containing all necessary steps (pipetting, washing, plate movement, etc.) in a loop. A new iteration of this loop is started as soon as a new handshake file is written by the supervising software. After completion of all LHS steps, the handshake file is deleted as the last step in the loop, which is the signal for the supervising software to continue with MBR cultivation.

Automated harvest of MBR cultures was based on the dynamics of the DO signal monitored online to detect the end of the exponential growth phase by a sharp increase in the DO signal. This detection was implemented by two sequential conditions that had to be fulfilled. First, the DO signal had to fall below 50% air saturation (a.s.). After that, the DO had to rise above 80% a.s., which is the trigger condition to cause the pipetting of cell suspension from the BioLector device. The supervising software thus ordered the cover of the BioLector’s incubation chamber to be opened and the LHS to aspirate the cell suspension 1 mm above the bottom of the cultivation microtitre plate (MTP) placed in the BioLector device. Per culture, 700 µl cell suspension was removed and pipetted into a 96‐deep‐well plate (Riplate, Ritter, Schwabmünchen, Germany). The cover of the BioLector was then closed, and the next harvesting cycle was initiated. In parallel, the 96‐deep‐well plate containing the cell suspensions was placed into an LHS accessible centrifuge (Ixion, SIAS, Hombrechtikon, Switzerland) to pellet the cells for 10 min at 2000 *g*. The resulting supernatants were subsequently transferred to another 96‐deep‐well plate, covered with a self‐adhesive aluminium foil (SILVERseal, Sigma‐Aldrich, Steinheim, Germany) and cooled to 4 °C. After completion of the MBR cultivation experiment, supernatants were stored at −20 °C until required for analysis.

### Fed‐batch bioreactor cultivations

Cultivations in stirred tank reactors (STR) with four biological replicates were conducted according to Hemmerich and colleagues (2019a) in a two‐phase process with a non‐induced initial batch phase, followed by an induced (50 µM IPTG) glucose‐limited fed‐batch phase with a glucose feeding profile, using a feeding profile parameter of *µ*_Set_ = 0.15 h^−1^. Frozen WCB strain aliquots (5 ml) for STR cultivations were produced as described previously (Hemmerich *et al*., 2019a) and used for bioreactor inoculation at a starting volume of 800 ml.

### Analysis

#### Biomass concentration

Optical density was measured at 600 nm (OD_600_) against PBS. Samples were diluted with PBS to a range of approximately 0.05–0.5 OD_600_. Cell dry weight was determined gravimetrically, for which purpose samples of 1 ml were pipetted into pre‐dried (80 °C, > 48 h) and pre‐weighed 2‐ml tubes and centrifuged at maximum speed for 10 min in a benchtop centrifuge (Biofuge Pico, Heraeus, Hanau, Germany). Cell pellets were washed once with PBS and centrifuged again, and the supernatant was discarded. The pellets were dried as before with subsequent weighing to calculate the cell dry weight *c*_X_ [g_X_ l^−1^]. To convert backscatter measurements from the BioLector MBR cultivations into cell dry weight, calibration series were recorded for different *C. glutamicum* strains. To this end, a sufficient amount of cell suspension was produced from 1000‐ml shake flask cultivations in CGXII medium and the resulting biomass was washed and resuspended to different cell concentrations in CGXII medium without glucose. For each dilution step, a cell dry weight from 2 or 10 ml of cell suspension was determined in three analytical replicates, and backscatter was determined in three technical replicates by filling three times 800 µl of each dilution step into a FlowerPlate, which was measured in the BioLector devices using the cultivation protocol. Values for backscatter and cell dry weight were then correlated by linear regression.

#### Cutinase activity assay

Cutinase activity from cultivation supernatants, stored at −20 °C, was determined using *p*‐nitrophenylpalmitate (pNPP) as the substrate analogon with the *p*‐nitrophenol (pNP) anion as the spectrophotometrically detectable reaction product (Winkler and Stuckmann, 1979). Samples were diluted appropriately using PBS, and 20 µl of the diluted samples was copied into three 96‐well MTPs, serving as analytical triplicates for cutinase activity measurements. In each MTP, three replicates of a pNP dilution series were pipetted (20 µL per dilution step) to convert absorption readings at 410 nm into micromoles of pNP formed during pNPP hydrolysis by cutinase. The enzymatic reaction was started by the rapid addition of 200 µl reaction mix, MTPs were subsequently transferred into an MTP reader pre‐warmed to 37 °C, and absorption at 410 nm was recorded at 25 s intervals. The reaction mix consisted of 1 volume of substrate solution (30 mg pNPP in 10 ml isopropanol) and 10 volumes of reaction buffer (2.3 g l^−1^ Na‐deoxycholate, 1.1 g l^−1^ gum arabic in 55 mM K‐P_i_ buffer, pH 8). The resulting linear increase over time Δ*A*_410_ [a.u. min^−1^], R^2^> 0.99, multiplied by the sample dilution factor and the slope of the pNP standards vs. absorption readings *a*_pNP_ [µmol l^−1^ a.u.^‐1^], was used to determine cutinase activity EA [U l^−1^]. Supernatants of *C. glutamicum* strains without plasmid for cutinase expression and secretion showed no cutinase enzyme activity (data not shown).

### Calculation of growth rates and cutinase yields

For MBR cultivations, growth rates *µ* [h^−1^] were calculated as described (Hemmerich *et al*., 2017) and biomass‐specific cutinase yields *Y*_P/X_ [kU g_X_^−1^] were calculated as a ratio of cutinase activity EA [U l^−1^] and cell dry weight *c*_X_ [g_X_ l^−1^], as determined from backscatter measurements at the time of harvesting. Observed growth (*µ*_Exp_) rates and yields from fed‐batch bioreactor cultivations were calculated as described elsewhere (Hemmerich *et al*., 2019a). For modified and/or plasmid‐containing strains, the observed growth rate *µ*_Exp_ will differ from the value of the technical parameter *µ*_Set_ needed to define the feeding protocol (Hemmerich *et al*., 2019a). For strains WT_NprE and GRS41_51_NprE, substrate‐specific cutinase yields *Y*_P/S_ [kU g_S_^−1^] have been calculated from MBR batch cultivations as a ratio of cutinase activity and consumed glucose concentration at the time of harvesting, if available. For STR fed‐batch cultivations, values of *Y*_P/S_ were obtained by linear regression from consumed glucose and cutinase activity, using eight sampling time points from the fed‐batch phase of each cultivation, as described for the determination of *Y*_P/X_ (Hemmerich *et al*., 2019a).

## Conflict of interest

The authors declare that they have no conflict of interest.

## Supporting information


**Table S1.** Summary of results from MBR quantitative phenotyping of *C. glutamicum* GRS library with either AmyE SP or NprE SP for cutinase secretion.
**Table S2.** Annotations for deleted genes in GRS41_51_NprE, according to Baumgart *et al*. 2018.
**Table S3.** Oligonucleotides used in this study.

## References

[mbt213660-bib-0001] Baumgart, M. , Unthan, S. , Kloß, R. , Radek, A. , Polen, T. , Tenhaef, N. , *et al* (2018) *Corynebacterium glutamicum* chassis C1*: Building and testing a novel platform host for synthetic biology and industrial biotechnology. ACS Synth Biol 7: 132–144. 28803482 10.1021/acssynbio.7b00261

[mbt213660-bib-0002] Baumgart, M. , Unthan, S. , Rückert, C. , Sivalingam, J. , Grünberger, A. , Kalinowski, J. , *et al* (2013) Construction of a prophage‐free variant of *Corynebacterium glutamicum* ATCC 13032 for use as a platform strain for basic research and industrial biotechnology. Appl Environ Microbiol 79: 6006–6015. 23892752 10.1128/AEM.01634-13 PMC3811366

[mbt213660-bib-0003] Brockmeier, U. , Caspers, M. , Freudl, R. , Jockwer, A. , Noll, T. , and Eggert, T. (2006) Systematic screening of all signal peptides from *Bacillus subtilis*: a powerful strategy in optimizing heterologous protein secretion in Gram‐positive bacteria. J Mol Biol 362: 393–402. 16930615 10.1016/j.jmb.2006.07.034

[mbt213660-bib-0004] Chen, S. , Su, L. , Chen, J. , and Wu, J. (2013) Cutinase: characteristics, preparation, and application. Biotechnol Adv 31: 1754–1767. 24055682 10.1016/j.biotechadv.2013.09.005

[mbt213660-bib-0005] Choe, D. , Cho, S. , Kim, S.C. , and Cho, B.‐K. (2016) Minimal genome: worthwhile or worthless efforts toward being smaller? Biotechnol J 11: 199–211. 26356135 10.1002/biot.201400838

[mbt213660-bib-0006] Cruz Bournazou, M.N. , Barz, T. , Nickel, D.B. , Lopez Cárdenas, D.C. , Glauche, F. , Knepper, A. , and Neubauer, P. (2017) Online optimal experimental re‐design in robotic parallel fed‐batch cultivation facilities. Biotechnol Bioeng 114: 610–619. 27696353 10.1002/bit.26192

[mbt213660-bib-0007] Flitsch, D. , Krabbe, S. , Ladner, T. , Beckers, M. , Schilling, J. , Mahr, S. , *et al* (2016) Respiration activity monitoring system for any individual well of a 48‐well microtiter plate. J Bio Eng 10: 14. 27795735 10.1186/s13036-016-0034-3 PMC5081973

[mbt213660-bib-0008] Forster, A.C. , and Church, G.M. (2006) Towards synthesis of a minimal cell. Mol Syst Biol 2: 45. 16924266 10.1038/msb4100090 PMC1681520

[mbt213660-bib-0009] Freudl, R. (2017) Beyond amino acids: Use of the *Corynebacterium glutamicum* cell factory for the secretion of heterologous proteins. J Biotechnol 258: 101–109. 28238807 10.1016/j.jbiotec.2017.02.023

[mbt213660-bib-0010] Hemmerich, J. , Moch, M. , Jurischka, S. , Wiechert, W. , Freudl, R. , and Oldiges, M. (2019a) Combinatorial impact of Sec signal peptides from *Bacillus subtilis* and bioprocess conditions on heterologous cutinase secretion by *Corynebacterium glutamicum* . Biotechnol Bioeng 116: 644–655. 30450544 10.1002/bit.26873

[mbt213660-bib-0011] Hemmerich, J. , Noack, S. , Wiechert, W. , and Oldiges, M. (2018) Microbioreactor systems for accelerated bioprocess development. Biotechnol J 13: e1700141. 29283217 10.1002/biot.201700141

[mbt213660-bib-0012] Hemmerich, J. , Tenhaef, N. , Steffens, C. , Kappelmann, J. , Weiske, M. , Reich, S.J. , *et al* (2019b) Less sacrifice, more insight: Repeated low‐volume sampling of microbioreactor cultivations enables accelerated deep phenotyping of microbial strain libraries. Biotechnol J 14: e1800428. 30318833 10.1002/biot.201800428

[mbt213660-bib-0013] Hemmerich, J. , Wiechert, W. , and Oldiges, M. (2017) Automated growth rate determination in high‐throughput microbioreactor systems. BMC Res Notes 10: 617. 29178966 10.1186/s13104-017-2945-6 PMC5702135

[mbt213660-bib-0014] Hohmann, H.‐P. , van Dijl, J.M. , Krishnappa, L. , and Prágai, Z. (2017) Host organisms: *Bacillus subtilis* In Industrial Biotechnology: Microorganisms. Wittmann C., and Liao J. (eds). Weinheim, Germany: Wiley‐VCH, pp. 221–298.

[mbt213660-bib-0015] Huber, R. , Ritter, D. , Hering, T. , Hillmer, A.‐K. , Kensy, F. , Müller, C. , *et al* (2009) Robo‐Lector – a novel platform for automated high‐throughput cultivations in microtiter plates with high information content. Microb Cell Fact 8: 42. 19646274 10.1186/1475-2859-8-42 PMC2731075

[mbt213660-bib-0016] Juhas, M. , Reuß, D.R. , Zhu, B. , and Commichau, F.M. (2014) *Bacillus subtilis* and *Escherichia coli* essential genes and minimal cell factories after one decade of genome engineering. Microbiology 160: 2341–2351. 25092907 10.1099/mic.0.079376-0

[mbt213660-bib-0017] Käß, F. , Hariskos, I. , Michel, A. , Brandt, H.‐J. , Spann, R. , Junne, S. , *et al* (2014) Assessment of robustness against dissolved oxygen/substrate oscillations for *C. glutamicum* DM1933 in two‐compartment bioreactor. Bioprocess Biosyst Eng 37: 1151–1162. 24218302 10.1007/s00449-013-1086-0

[mbt213660-bib-0018] Keilhauer, C. , Eggeling, L. , and Sahm, H. (1993) Isoleucine synthesis in *Corynebacterium glutamicum*: Molecular analysis of the *ilvB*‐*ilvN*‐*ilvC* operon. J Bacteriol 175: 5595–5603. 8366043 10.1128/jb.175.17.5595-5603.1993 PMC206616

[mbt213660-bib-0019] Kensy, F. , Zang, E. , Faulhammer, C. , Tan, R.‐K. , and Büchs, J. (2009) Validation of a high‐throughput fermentation system based on online monitoring of biomass and fluorescence in continuously shaken microtiter plates. Microb Cell Fact 8: 31. 19497126 10.1186/1475-2859-8-31 PMC2700080

[mbt213660-bib-0020] Kinoshita, S. , Udaka, S. , and Shimono, M. (1957) Studies on the amino acid fermentation. Part 1. Production of L‐glutamic acid by various microorganisms. J Gen Appl Microbiol 3: 193–205. 15965888

[mbt213660-bib-0021] Knoll, A. , Bartsch, S. , Husemann, B. , Engel, P. , Schroer, K. , Ribeiro, B. , *et al* (2007) High cell density cultivation of recombinant yeasts and bacteria under non‐pressurized and pressurized conditions in stirred tank bioreactors. J Biotechnol 132: 167–179. 17681630 10.1016/j.jbiotec.2007.06.010

[mbt213660-bib-0022] Ladner, T. , Beckers, M. , Hitzmann, B. , and Büchs, J. (2016) Parallel online multi‐wavelength (2D) fluorescence spectroscopy in each well of a continuously shaken microtiter plate. Biotechnol J 11: 1605–1616. 27860327 10.1002/biot.201600515

[mbt213660-bib-0023] Lattermann, C. , and Büchs, J. (2015) Microscale and miniscale fermentation and screening. Curr Opin Biotechnol 35: 1–6. 25544012 10.1016/j.copbio.2014.12.005

[mbt213660-bib-0024] Lieder, S. , Nikel, P.I. , de Lorenzo, V. , and Takors, R. (2015) Genome reduction boosts heterologous gene expression in *Pseudomonas putida* . Microb Cell Fact 14: 23. 25890048 10.1186/s12934-015-0207-7 PMC4352270

[mbt213660-bib-0025] Limberg, M.H. , Pooth, V. , Wiechert, W. , and Oldiges, M. (2016) Plug flow versus stirred tank reactor flow characteristics in two‐compartment scale‐down bioreactor: Setup‐specific influence on the metabolic phenotype and bioprocess performance of *Corynebacterium glutamicum* . Eng Life Sci 16: 610–619.

[mbt213660-bib-0026] Limberg, M.H. , Schulte, J. , Aryani, T. , Mahr, R. , Baumgart, M. , Bott, M. , *et al* (2017) Metabolic profile of 1,5‐diaminopentane producing *Corynebacterium glutamicum* under scale‐down conditions: Blueprint for robustness to bioreactor inhomogeneities. Biotechnol Bioeng 114: 560–575. 27641904 10.1002/bit.26184

[mbt213660-bib-0027] Liu, L. , Yang, H. , Shin, H.‐D. , Chen, R.R. , Li, J. , Du, G. , and Chen, J. (2013a) How to achieve high‐level expression of microbial enzymes: Strategies and perspectives. Bioengineered 4: 212–223. 23686280 10.4161/bioe.24761 PMC3728192

[mbt213660-bib-0028] Liu, L. , Yang, H. , Shin, H.‐D. , Li, J. , Du, G. , and Chen, J. (2013b) Recent advances in recombinant protein expression by *Corynebacterium*, *Brevibacterium*, and *Streptomyces*: From transcription and translation regulation to secretion pathway selection. Appl Microbiol Biotechnol 97: 9597–9608. 24068337 10.1007/s00253-013-5250-x

[mbt213660-bib-0029] Looser, V. , Bruhlmann, B. , Bumbak, F. , Stenger, C. , Costa, M. , Camattari, A. , *et al* (2015) Cultivation strategies to enhance productivity of *Pichia pastoris*: a review. Biotechnol Adv 33: 1177–1193. 26027890 10.1016/j.biotechadv.2015.05.008

[mbt213660-bib-0030] Martínez‐García, E. , and de Lorenzo, V. (2016) The quest for the minimal bacterial genome. Curr Opin Biotechnol 42: 216–224. 27660908 10.1016/j.copbio.2016.09.001

[mbt213660-bib-0031] Mathiesen, G. , Sveen, A. , Brurberg, M.B. , Fredriksen, L. , Axelsson, L. , and Eijsink, V.G. (2009) Genome‐wide analysis of signal peptide functionality in *Lactobacillus plantarum* WCFS1. BMC Genom 10: 425. 10.1186/1471-2164-10-425 PMC2748100 19744343

[mbt213660-bib-0032] Neubauer, P. , Cruz, N. , Glauche, F. , Junne, S. , Knepper, A. , and Raven, M. (2013) Consistent development of bioprocesses from microliter cultures to the industrial scale. Eng Life Sci 13: 224–238.

[mbt213660-bib-0033] Noack, S. , and Baumgart, M. (2019) Communities of niche‐optimized strains: Small‐genome organism consortia in bioproduction. Trends Biotechnol 37: 126–139. 30115374 10.1016/j.tibtech.2018.07.011

[mbt213660-bib-0034] Pósfai, G. , Plunkett, G. , Fehér, T. , Frisch, D. , Keil, G.M. , Umenhoffer, K. , *et al* (2006) Emergent properties of reduced‐genome *Escherichia coli* . Science 312: 1044–1046. 16645050 10.1126/science.1126439

[mbt213660-bib-0035] Ravasi, P. , Braia, M. , Eberhardt, F. , Elena, C. , Cerminati, S. , Peirú, S. , *et al* (2015) High‐level production of *Bacillus cereus* phospholipase C in *Corynebacterium glutamicum* . J Biotechnol 216: 142–148. 26519562 10.1016/j.jbiotec.2015.10.018

[mbt213660-bib-0036] van der Rest, M.E. , Lange, C. , and Molenaar, D. (1999) A heat shock following electroporation induces highly efficient transformation of *Corynebacterium glutamicum* with xenogeneic plasmid DNA. Appl Microbiol Biotechnol 52: 541–545. 10570802 10.1007/s002530051557

[mbt213660-bib-0037] Reuß, D.R. , Altenbuchner, J. , Mäder, U. , Rath, H. , Ischebeck, T. , Sappa, P.K. , *et al* (2017) Large‐scale reduction of the *Bacillus subtilis* genome: Consequences for the transcriptional network, resource allocation, and metabolism. Genome Res 27: 289–299. 27965289 10.1101/gr.215293.116 PMC5287234

[mbt213660-bib-0038] Riesenberg, D. , and Guthke, R. (1999) High‐cell‐density cultivation of microorganisms. Appl Microbiol Biotechnol 51: 422–430. 10341426 10.1007/s002530051412

[mbt213660-bib-0039] Rohe, P. , Venkanna, D. , Kleine, B. , Freudl, R. , and Oldiges, M. (2012) An automated workflow for enhancing microbial bioprocess optimization on a novel microbioreactor platform. Microb Cell Fact 11: 144. 23113930 10.1186/1475-2859-11-144 PMC3526558

[mbt213660-bib-0040] Schäfer, A. , Tauch, A. , Jäger, W. , Kalinowski, J. , Thierbach, G. , and Pühler, A. (1994) Small mobilizable multi‐purpose cloning vectors derived from the *Escherichia coli* plasmids pK18 and pK19: Selection of defined deletions in the chromosome of *Corynebacterium glutamicum* . Gene 145: 69–73. 8045426 10.1016/0378-1119(94)90324-7

[mbt213660-bib-0041] Sharma, S.S. , Blattner, F.R. , and Harcum, S.W. (2007) Recombinant protein production in an *Escherichia coli* reduced genome strain. Metab Eng 9: 133–141. 17126054 10.1016/j.ymben.2006.10.002 PMC3710453

[mbt213660-bib-0042] Tanaka, K. , Henry, C.S. , Zinner, J.F. , Jolivet, E. , Cohoon, M.P. , Xia, F. , *et al* (2013) Building the repertoire of dispensable chromosome regions in *Bacillus subtilis* entails major refinement of cognate large‐scale metabolic model. Nucleic Acids Res 41: 687–699. 23109554 10.1093/nar/gks963 PMC3592452

[mbt213660-bib-0043] Unthan, S. , Baumgart, M. , Radek, A. , Herbst, M. , Siebert, D. , Brühl, N. , *et al* (2015a) Chassis organism from *Corynebacterium glutamicum* – a top‐down approach to identify and delete irrelevant gene clusters. Biotechnol J 10: 290–301. 25139579 10.1002/biot.201400041 PMC4361050

[mbt213660-bib-0044] Unthan, S. , Radek, A. , Wiechert, W. , Oldiges, M. , and Noack, S. (2015b) Bioprocess automation on a Mini Pilot Plant enables fast quantitative microbial phenotyping. Microb Cell Fact 14: 32. 25888907 10.1186/s12934-015-0216-6 PMC4361198

[mbt213660-bib-0045] Watanabe, K. , Teramoto, H. , Suzuki, N. , Inui, M. , and Yukawa, H. (2013) Influence of SigB inactivation on *Corynebacterium glutamicum* protein secretion. Appl Microbiol Biotechnol 97: 4917–4926. 23179627 10.1007/s00253-012-4586-y

[mbt213660-bib-0046] Watanabe, K. , Tsuchida, Y. , Okibe, N. , Teramoto, H. , Suzuki, N. , Inui, M. , and Yukawa, H. (2009) Scanning the *Corynebacterium glutamicum* R genome for high‐efficiency secretion signal sequences. Microbiology 155: 741–750. 19246745 10.1099/mic.0.024075-0

[mbt213660-bib-0047] Wendisch, V.F. (2014) Microbial production of amino acids and derived chemicals: synthetic biology approaches to strain development. Curr Opin Biotechnol 30: 51–58. 24922334 10.1016/j.copbio.2014.05.004

[mbt213660-bib-0048] Wendisch, V.F. , Jorge, J.M.P. , Pérez‐García, F. , and Sgobba, E. (2016) Updates on industrial production of amino acids using *Corynebacterium glutamicum* . World J Microbiol Biotechnol 32: 105. 27116971 10.1007/s11274-016-2060-1

[mbt213660-bib-0049] Winkler, U.K. , and Stuckmann, M. (1979) Glycogen, hyaluronate, and some other polysaccharides greatly enhance the formation of exolipase by *Serratia marcescens* . J Bacteriol 138: 663–670. 222724 10.1128/jb.138.3.663-670.1979 PMC218088

[mbt213660-bib-0050] Yee, L. , and Blanch, H.W. (1992) Recombinant protein expression in high cell density fed‐batch cultures of *Escherichia coli* . Nat Biotechnol 10: 1550–1556. 10.1038/nbt1292-1550 1369204

[mbt213660-bib-0051] Yim, S.S. , An, S.J. , Kang, M. , Lee, J. , and Jeong, K.J. (2013) Isolation of fully synthetic promoters for high‐level gene expression in *Corynebacterium glutamicum* . Biotechnol Bioeng 110: 2959–2969. 23633298 10.1002/bit.24954

[mbt213660-bib-0052] Yim, S.S. , Choi, J.W. , Lee, R.J. , Lee, Y.J. , Lee, S.H. , Kim, S.Y. , and Jeong, K.J. (2016) Development of a new platform for secretory production of recombinant proteins in *Corynebacterium glutamicum* . Biotechnol Bioeng 113: 163–172. 26134574 10.1002/bit.25692

[mbt213660-bib-0053] Zhang, B. , Zhou, N. , Liu, Y.‐M. , Liu, C. , Lou, C.‐B. , Jiang, C.‐Y. , and Liu, S.‐J. (2015) Ribosome binding site libraries and pathway modules for shikimic acid synthesis with *Corynebacterium glutamicum* . Microb Cell Fact 14: 71. 25981633 10.1186/s12934-015-0254-0 PMC4453273

