# Supplementary material for: Screening of a genome‐reduced Corynebacterium glutamicum strain library for improved heterologous cutinase secretion
Source: Microb Biotechnol. 2020 Sep 6;13(6):2020–31. doi: 10.1111/1751-7915.13660 (PMC7533341; doi:10.1111/1751-7915.13660)
Supplement: Supplementary file 1 — Table S1. Summary of results from MBR quantitative phenotyping of C. glutamicum GRS library with either AmyE SP or NprE SP for cutinase secretion. Table S2. Annotations for deleted genes in GRS41_51_NprE, according to Baumgart et al. 2018. Table S3. Oligonucleotides used in this study. [file MBT2-13-2020-s001.pdf]

Supporting information for research article

Screening of a genome-reduced *Corynebacterium glutamicum* strain library for improved heterologous cutinase secretion

Johannes Hemmerich[1,6], Mohamed Labib[1], Carmen Steffens[1], Sebastian J. Reich[1,§], Marc Weiske[1], Meike Baumgart[1], Christian Rückert[2], Matthias Ruwe[2], Daniel Siebert[3,$], Volker F. Wendisch[3], Jörn Kalinowski[2], Wolfgang Wiechert[1,4,6], and Marco Oldiges[1,5,6,#]

[1]: Forschungszentrum Jülich, Institute of Bio- and Geosciences - Biotechnology (IBG-1), 52425 Jülich/DE

[2]: Microbial Genomics and Biotechnology, Center for Biotechnology, Bielefeld University, 33615 Bielefeld/DE

[3]: Chair of Genetics of Prokaryotes, Faculty of Biology, Bielefeld University, 33615 Bielefeld/DE

[4]: RWTH Aachen University, Computational Systems Biotechnology (AVT.CSB), 52074 Aachen/DE

[5]: RWTH Aachen University, Institute of Biotechnology, 52074 Aachen/DE

[6]: Bioeconomy Science Center (BioSC), c/o Forschungszentrum Jülich, 52425 Jülich/DE

[§]: Current affiliation: Institute of Microbiology and Biotechnology, Ulm University, 89081 Ulm/DE

[$]: Current affiliation: Microbial Biotechnology, Campus Straubing for Biotechnology and Sustainability, Technical University of Munich, 94315 Straubing/DE

[#]: Correspondence: m.oldiges@fz-juelich.de

Table S1: Summary of results from MBR quantitative phenotyping of *C. glutamicum* GRS library with either AmyE SP or NprE SP for cutinase secretion.

	**AmyE SP**			**NprE SP**		
**Background strain**	μ **[h^{-1}]**	$Y_{P/X}$ **[kU g$_X^{-1}$]**	***n***	μ **[h^{-1}]**	$Y_{P/X}$ **[kU g$_X^{-1}$]**	***n***
WT	0.42 ± 0.01	0.23 ± 0.04	20	0.40 ± 0.02	0.53 ± 0.13	46
MB001	0.39 ± 0.01	0.29 ± 0.09	12	0.33 ± 0.02	0.52 ± 0.10	26
GRS21	0.39 ± 0.01	0.30 ± 0.04	12	0.33 ± 0.02	0.49 ± 0.09	33
GRS25	0.29 ± 0.01	0.37 ± 0.12	12	0.28 ± 0.01	0.51 ± 0.12	12
GRS41	0.32 ± 0.01	0.35 ± 0.07	12	0.30 ± 0.01	0.69 ± 0.14	27
GRS45	0.30 ± 0.01	0.29 ± 0.07	12	0.16 ± 0.01	0.74 ± 0.09	9
GRS51	0.34 ± 0.01	0.28 ± 0.04	12	0.20 ± 0.01	0.82 ± 0.22	26
GRS53	0.40 ± 0.01	0.24 ± 0.07	14	0.37 ± 0.03	0.56 ± 0.10	22
GRS55	0.30 ± 0.01	0.29 ± 0.06	12	0.26 ± 0.01	0.71 ± 0.09	11
GRS56	0.38 ± 0.01	0.45 ± 0.15	12	0.34 ± 0.02	0.49 ± 0.22	26
GRS21_41	0.31 ± 0.01	0.40 ± 0.04	12	0.27 ± 0.01	0.88 ± 0.15	28
GRS41_51	0.30 ± 0.01	0.31 ± 0.05	12	0.15 ± 0.01	1.62 ± 0.26	23
GRS48_52	0.32 ± 0.01	0.28 ± 0.05	14	0.23 ± 0.03	0.25 ± 0.10	25
GRS56_57	0.38 ± 0.01	0.39 ± 0.09	12	0.33 ± 0.01	0.63 ± 0.27	15
GRS56_58	0.37 ± 0.01	0.44 ± 0.09	12	0.32 ± 0.02	0.51 ± 0.22	16
GRS57_58	0.36 ± 0.02	0.24 ± 0.03	11	0.33 ± 0.03	0.36 ± 0.14	17
GRS48_52_53	0.33 ± 0.01	0.24 ± 0.04	14	0.18 ± 0.01	1.29 ± 0.25	36
GRS56_57_58	0.34 ± 0.02	0.24 ± 0.04	11	0.31 ± 0.02	0.40 ± 0.12	26
PC1	0.42 ± 0.01	0.29 ± 0.06	14	0.34 ± 0.03	0.65 ± 0.13	22
PC1_53	0.41 ± 0.01	0.23 ± 0.04	12	0.34 ± 0.02	0.78 ± 0.15	14
PC2_29	0.19 ± 0.03	0.01 ± 0.01	10	0.26 ± 0.01	0.69 ± 0.17	18
C1	0.39 ± 0.01	0.34 ± 0.09	12	0.33 ± 0.01	0.55 ± 0.08	12
C2	0.34 ± 0.01	0.30 ± 0.08	11	0.33 ± 0.01	0.47 ± 0.06	12

Results are given as mean with standard deviation from *n* replicate cultivations, as indicated, and originate from two to five independently carried out MBR growth experiments. Growth rates and cutinase yields for strains WT_NprE, GRS53_NprE, GRS48_52_NprE, GRS48_52_53_NprE, PC1_NprE and PC1_53_NprE include previously reported data (Hemmerich et al. 2019b). μ, growth rate. $Y_{P/X}$, biomass specific cutinase yield. *GRS*, genome-reduced strain. *MBR*, microbioreactor. *SP*, signal peptide. *n*, number of cultivation replicates.

Table S2: Annotations for deleted genes in GRS41_51_NprE, according to Baumgart et al. 2018.

Cg no.	Gene	Protein	Annotation, additional information
cg2801	*ccrB*	CrcB	camphor resistance protein CrcB, N-terminal fragment, putative pseudogene
cg2802	*crcB*	CrcB	membrane protein similar to CrcB, involved into chromosome folding C-terminal fragment, putative pseudogene
cg2803			hypothetical protein, conserved
cg2804	*tnp21a*	ISCg21a	transposase
cg2805	*psp4*	Psp4	putative secreted protein
cg2806			putative membrane protein
cg2807	*tnp11a*	ISCg11a	transposase, putative pseudogene
cg2808	*tnp13a*	ISCg13a	transposase
cg2809			putative membrane protein
cg2810	*cynT*	CynT	high affinity cysteine importer
cg2811			putative ABC-type transport system, involved in lipoprotein release, permease component
cg2812			putative ABC-type transport system, involved in lipoprotein release, ATPase component
cgr10			5S ribosomal RNA
cgr11			23S ribosomal RNA
cgr12			16S ribosomal RNA
cg2822			putative sugar phosphate isomerase/epimerase
cg2823			putative dehydrogenase or related protein
cg2824			putative SAM-dependent methyltransferase
cgr13			5S ribosomal RNA
cgr14			23S ribosomal RNA
cgr15			16S ribosomal RNA
cg2828			putative membrane protein
cgr16			5S ribosomal RNA
cgr17			23S ribosomal RNA
cgr18			16S ribosomal RNA
cg3261			putative transcriptional regulator, GntR-family
cg3263			hypothetical protein, conserved
cg3264	*rsmP*	RsmP	cytoskeletal protein RsmP, regulates rod-shape morphology, conserved
cg3266	*tnp5c*	ISCg5c	transposase
cg3267			putative membrane protein, putative pseudogene, C-terminal fragment
cg3268			putative membrane protein, putative pseudogene
cg3269			putative membrane protein, putative pseudogene
cg3270			putative membrane protein, putative pseudogene, N-terminal fragment
cg3271			putative SAM-dependent methyltransferase
cg3272			putative membrane protein
cg3273			hypothetical protein
cg3274			putative site-specific recombinase, DNA invertase Pin homolog-fragment, putative pseudogene
cg3275	*fdxA*	FdxA	ferredoxin no. 1
cg3277			putative protein, ACR, double-stranded β-helix domain
cg3278	*tnp20a*	ISCg20a	transposase, putative pseudogene
cg3279			putative dehydrogenase-fragment, putative pseudogene
cg3280			putative secreted protein, horizontally transferred gene
cg3281	*copB*	CopB	Cu^{2+}/cation-transporting ATPase transmembrane protein, horizontally transferred gene
cg3282			putative Cu^{2+}/heavy metal binding transport protein, horizontally transferred gene
cg3283			hypothetical protein, horizontally transferred gene
cg3284	*copS* (*cgtS9*)	CopS	two component sensor kinase, copper homeostasis, horizontally transferred gene
cg3285	*copR* (*cgtR9*)	CopR	two component response regulator, copper homeostasis, horizontally transferred gene

cg3286			putative secreted protein of unknown function, horizontally transferred gene
cg3287	*copO*	CopO	secreted multicopper oxidase, horizontally transferred gene
cg3288			hypothetical protein, horizontally transferred gene
cg3289	*tlpA*	TlpA	thioredoxin-like protein no. 4, horizontally transferred gene
cg3290			putative oxidoreductase protein, horizontally transferred gene
cg3291			putative transcriptional regulator, Crp-family, horizontally transferred gene
cg3292			putative copper chaperone, horizontally transferred gene
cg3293			hypothetical protein, horizontally transferred gene
cg3294			hypothetical protein, horizontally transferred gene
cg3295			putative Cd^{2+}/cation transport ATPase, horizontally transferred gene
cg3296	*tnp19c*	ISCg19a	transposase fragment, putative pseudogene
cg3297	*tnp19b*	ISCg19a	transposase fragment, putative pseudogene
cg3298	*tnp19a*	ISCg19a	transposase fragment, putative pseudogene

Table S3: Oligonucleotides used in this study.

Oligonucleotide	Sequence (5' → 3') and properties[a]
Construction of deletion plasmid pK19mobsacB-Δcg3000-3006 and PCR-analysis of the resulting mutants	
cg3000-06-D1	CCGGAATTCATCAACGGTGACTTGGCGTG
cg3000-06-D2	**CCCATCCACTAAACTTAAACA**GTTTGTCCCCACAGGCTAGTC
cg3000-06-D3	**TGTTTAAGTTTAGTGGATGGG**TACTGAAAGGTGCTGGGGAG
cg3000-06-D4	CGCGGATCCTTGACCGCTCTTGGTACAACC
cg3000-06-Dfw	CACTGCTGATCCAGTAGCAAG
cg3000-06-Drv	GTACTTCCAGCGCTATATTCAGC
PCR-analysis of the resulting mutants from deletion plasmid pK19mobsacBΔcg1370-cg1385	
cg1370_k1	CGCTTTCGAGCGTATTTG
cg1385_k2	AACCCGTCACGATGAGAG
PCR-analysis of the resulting mutants from deletion plasmid pK19mobsacBΔcg3324-cg3345	
MG5-Dfw	AACGTGGCGTTCGCGTAG
MG5-Drv	ACCGACGATGACAACAAGCAAC
Construction of deletion plasmid pK18mobsacB-ΔrrnB PCR-analysis of the resulting mutants	
pK18msB-ga1	AAGCGGAACACGTAGAAAGC
pK18msB-ga2	GGTGCCTAATGAGTGAGCTAAC
rrnB-D1	**CACTCATTAGGCACC**CGCTACATCGACTTCTTC
rrnB-D2	AAATCCGCAGGTTGAAGC
rrnB-D3	**TCAACCTGCGGATTT**GGATAAGCCCAGAAACAC
rrnB-D4	**CTACGTGTTCCGCTT**CGAATCAGGGTTGTACTC
rrnB-Dfw	CTGGCCAAGATCATCTCC
rrnB-Drv	AGCGACTCCAGATCTCAG
Construction of deletion plasmid pK18mobsacB-ΔrrnC and PCR-analysis of the resulting mutants	
pK18msB-ga1	AAGCGGAACACGTAGAAAGC
pK18msB-ga2	GGTGCCTAATGAGTGAGCTAAC
rrnC-D1	**CACTCATTAGGCACC**TTGTTTGCCCGACGTGAG
rrnC-D2	AAGTGCTCGAAGCGACAG
rrnC-D3	**TTTTCTGTCGCTTCGAGCACTT**GGGTTTAAGCGCTACTTG
rrnC-D4	**CTACGTGTTCCGCTT**GGCTCCTAATTCCGATTC
rrnC-Dfw	CGTGTGCTGACTGGAAAC
rrnC-Drv	AAGCTTGCGAGCAGGTAG
Construction of deletion plasmid pK18mobsacB-ΔrrnF and PCR-analysis of the resulting mutants	
pK18msB-ga1	AAGCGGAACACGTAGAAAGC
pK18msB-ga2	GGTGCCTAATGAGTGAGCTAAC
rrnF-D1	**CACTCATTAGGCACC**CGAGAATGCGTTGCAAGC
rrnF-D2	CGCTCGAGAAACTAGAAG
rrnF-D3	**AATACTTCTAGTTTCTCGAGCG**AGTAGGTTACCGCCGACC
rrnF-D4	**CTACGTGTTCCGCTT**GTAGCGGTCATTGCTACG
rrnF-Dfw	TAATCCCTCGTGGTGTTC
rrnF-Drv	TCATCACGTCGGTCATCG

[a] Overlaps for overlap extension PCR or Gibson assembly are written in bold letters.
